# Comparison of Motion Analysis Systems in Tracking Upper Body Movement of Myoelectric Bypass Prosthesis Users

**DOI:** 10.3390/s22082953

**Published:** 2022-04-12

**Authors:** Sophie L. Wang, Gene Civillico, Wesley Niswander, Kimberly L. Kontson

**Affiliations:** 1 Division of Biomedical Physics, Office of Science and Engineering Laboratories, Center for Devices and Radiological Health, Food and Drug Administration, Silver Spring, MD 20993, USA; swang9@terpmail.umd.edu (S.L.W.); wesniswander@gmail.com (W.N.); 2 Department of Bioengineering, University of Maryland, College Park, MD 20742, USA; 3 Institute for Chemical Imaging of Living Systems, Northeastern University, Boston, MA 02115, USA; e.civillico@northeastern.edu

**Keywords:** upper-limb, myoelectric, prosthesis, inertial measurement unit, Kinect, motion capture

## Abstract

Current literature lacks a comparative analysis of different motion capture systems for tracking upper limb (UL) movement as individuals perform standard tasks. To better understand the performance of various motion capture systems in quantifying UL movement in the prosthesis user population, this study compares joint angles derived from three systems that vary in cost and motion capture mechanisms: a marker-based system (Vicon), an inertial measurement unit system (Xsens), and a markerless system (Kinect). Ten healthy participants (5F/5M; 29.6 ± 7.1 years) were trained with a TouchBionic i-Limb Ultra myoelectric terminal device mounted on a bypass prosthetic device. Participants were simultaneously recorded with all systems as they performed standardized tasks. Root mean square error and bias values for degrees of freedom in the right elbow, shoulder, neck, and torso were calculated. The IMU system yielded more accurate kinematics for shoulder, neck, and torso angles while the markerless system performed better for the elbow angles. By evaluating the ability of each system to capture kinematic changes of simulated upper limb prosthesis users during a variety of standardized tasks, this study provides insight into the advantages and limitations of using different motion capture technologies for upper limb functional assessment.

## 1. Introduction

Motion analysis is a useful method to quantitatively and objectively assess human motion by providing kinematic information (e.g., joint angles, body trajectories, hand velocity, etc.) during task performance. A large proportion of studies investigating human motion use 3D optoelectric motion capture systems. These systems track the position of markers placed on anatomical landmarks of the body to relate the position and orientation of body segments. These systems are advantageous given their high resolution and accuracy, as well as their long history of use in research [1,2,3,4,5,6,7,8] compared to more recently developed mechanisms of motion capture [9,10,11,12,13,14]. While useful in many clinical populations, assessment of motion in the upper limb prosthesis user population is beneficial as the output of such analyses can aid in rehabilitation by providing more specific details about how a standard task is performed, as well as providing insights into the influence of upper limb prosthesis devices on motion. This is relevant given the upper limb prosthesis user population is known to employ compensatory movements during the performance of everyday tasks to work around lost degrees of freedom (DOF). Given recent technical developments in upper limb prosthesis devices with greater numbers of controllable DOFs [15,16,17], many research groups have investigated user movement with these devices using 3D optoelectric motion capture systems [16,17,18,19,20,21]. However, the adoption of 3D optoelectric motion capture into the clinic has been slow due to the restrictive operating environment required, high costs, and longer set-up times required to collect data from optoelectric motion capture systems [11,13]. 

Barriers to the use of optoelectric motion capture systems have prompted interest in other systems that have less restrictive operating environment requirements [22,23]. The Microsoft Kinect is a markerless motion capture sensor system that has been of great interest in research due to the low cost [23,24] and robustness of the sensors [11,13,22,25,26,27]. Due to the markerless motion capture mechanism, the set-up time is reduced and the potential for erroneous subject preparation is lower compared to marker-based motion capture systems that are dependent on accurate and consistent identification of anatomical landmarks. Alongside marker and markerless systems, battery and gyroscopic sensor miniaturization and the rapid decrease in technology costs has rendered inertial measurements a new avenue for motion capture research [28,29,30,31,32,33,34]. Due to the non-optical mechanism of inertial measurement unit (IMU) motion capture, the operating environment requirements are less restrictive compared to those required for optoelectric systems. 

There have been several previous studies comparing motion capture systems that have focused on one-to-one comparisons of a single test system and a gold standard system [29,32,35,36,37,38,39,40,41], studied the lower limbs [26,30,40,42,43,44,45], or relied on mechanical testing devices to ensure the greatest replicability of the ground truth [36,46,47,48]. For the one-to-one system comparisons, the parameters examined, motions selected, and populations tested varied greatly, rendering cross-system conclusions impractical. Regarding studies in the lower limb, the analyses lack applicability and generalizability to the tracking of motion in the upper limbs, specifically upper limb prosthesis users. Compared to the lower limbs, the acyclic motions and the multiple redundant DOFs in the upper limb make upper limb motion analysis more challenging. Furthermore, the few studies assessing upper limb function have focused on a limited task space to simplify capture and analysis [49]. Lastly, while the use of a mechanical testing device provides a highly consistent ground truth, it is not fully representative of system performance during human motion given the avoidance of soft tissue artifacts, sensor motion artifacts, and self-occlusion induced errors.

To better understand the performance of various motion capture systems in quantifying upper limb movement in the prosthesis user population, this study compares kinematics (i.e., joint angles) derived from three systems that vary in cost and motion capture mechanisms: a marker-based system, an IMU system, and a markerless system. Able-bodied individuals using a bypass prosthesis device performed several tasks as movement of the upper body was tracked simultaneously across all three systems. The results of this study can be used to identify consistencies and limitations of various motion capture systems in tracking movements similar to those performed by the upper limb prosthesis user population, which could facilitate the wider adoption of motion capture into rehabilitation. 

## 2. Materials and Methods

### 2.1. Participants

Ten able-bodied participants with no upper limb disability or impairment were included in this study. All subjects provided written informed consent prior to participating in the study. The study was conducted in accordance with the Declaration of Helsinki, and the protocol was approved by the U.S. FDA Institutional Review Board (Protocol #16-071). The participants were selected through convenience sampling (5 females, 5 males; mean age 29.6 ± 7.1 years). Nine of the ten participants were right-hand dominant (92.53 ± 10.62 laterality), one was left-hand dominant (−100 laterality) per the Edinburgh handedness survey [50]. 

**Figure 1:**
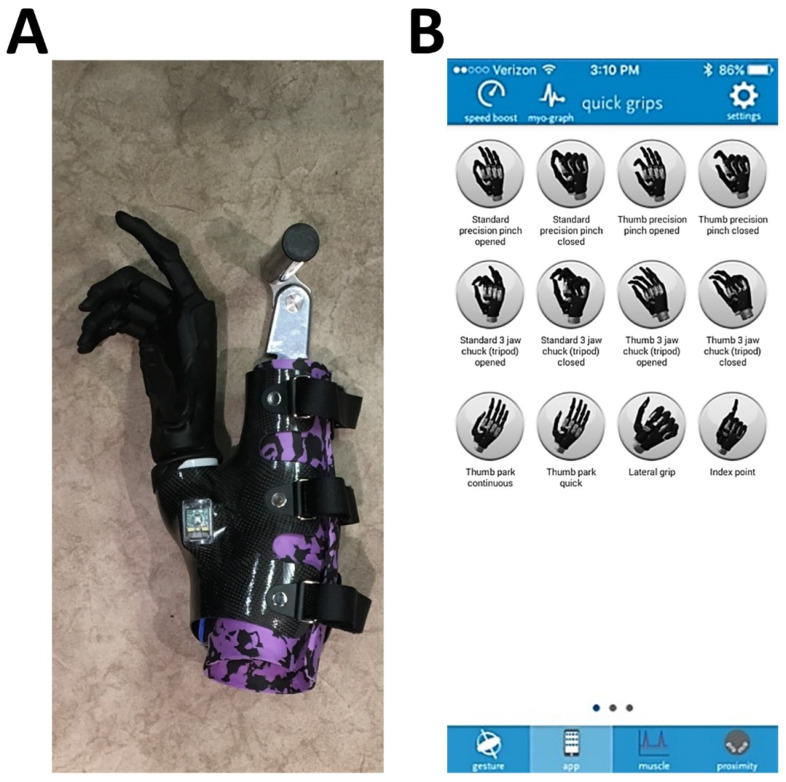
(**A**) Right-hand Ossur (TouchBionics) i-limb Ultra myoelectric terminal device. Medial offset = 15°; (**B**) my i-limb grip selection screenshot (Touch Bionics, Apple App Store, 2020).

A bypass prosthetic device was used by able-bodied individuals to elicit similar movement patterns as an upper limb prosthesis user [20,51,52,53,54,55]. A forearm brace adaptor with a perpendicular handlebar provided by Next Step Bionics allowed the use of a right-hand Ossur (previously TouchBionics) i-limb Ultra (OSSUR, Foothill Ranch, CA, USA) myoelectric terminal device with manual wrist adjustment. The device was mounted to the brace with a medial offset of 15° from the subject’s forearm (Figure 1A). In accordance with upper-limb amputee control configurations, myoelectric sensors were placed on antagonist pairs of extensor and flexor muscles on the forearm to control the opening and closing of the device. Grip changes were controlled with the TouchBionic my i-limb^TM^ app on an iPod Touch^TM^ (Apple Inc., Cupertino, CA, USA) (Figure 1B). This adaptive bypass device (MYO Bypass) allowed trained able-bodied participants to use a commercial upper limb prosthesis terminal device.

Following the protocol specified in Bloomer et al., 2019, all participants were trained with the right-hand MYO Bypass until a learning plateau of 90% peak performance had been achieved [56].

### 2.2. Functional Tasks

When fully trained, participants performed the Targeted Box and Blocks Test (tBBT) [57] as well as tasks selected from the Jebsen–Taylor Hand Function Test (JHFT) [58,59], Activities Measure for Upper Limb Amputees (AMULA) [60,61], and Comprehensive Assessment of Prosthetic Performance for Upper Limb (CAPPFUL) [62]. Outcome measures were simultaneously recorded by three motion analysis systems. With the motivation to determine the limitations of the motion analysis systems, tasks were selected from the outcome measures that would elicit a wide range of movements representative of those performed during activities of daily living. These outcome measures have also been used in previous kinematic studies [57,58,63] and most are validated in the upper limb prosthesis user population [62,64]. 

A brief description of each task used in this study can be found in Table 1. Tasks 2, 3, and 7 from the JHFT were performed in a seated position and are referenced in this manuscript as JHFT—Page Turn, JHFT—Small Objects, and JHFT—Heavy Objects, respectively. Tasks 10, 16, and 24 from the AMULA were performed in the seated position and are referenced as AMULA—Fork, AMULA—Doorknob, and AMULA—Reach, respectively [60,61]. Tasks 4, 8, and 11 from the CAPPFUL were also performed and are referenced as standing task CAPPFUL—Dice, and seated tasks CAPPFUL—Bottle, and CAPPFUL—Picture [62]. A standard template was used to place each object for a given task in the same location for each participant. The tBBT was performed in the standing position [57]. Participants performed three trials of each task. 

### 2.3. Motion Analysis Systems

Motion analysis involved the simultaneous recording of motion data from three systems: optical marker-based system (Vicon, Oxford, UK), an inertial measurement unit (IMU)-based system (Xsens Awinda MTw, El Segundo, CA, USA), and a markerless system (dual Microsoft Kinect V1s with iPI Recorder). The Vicon optical marker-based system was selected as the reference system based on its popularity and usage in the literature [27,36,37,38,44,45,48,65,66,67,68]. The IMU-based [28,29,30,31,32,39,40,41,42,43,69,70,71] and markerless systems [13,22,23,24,25,27,38,44,45,46,47,72,73] were selected due to popularity in the literature and due to their differing mechanisms of motion capture. Please see Appendix B for diagrams of the sensor placements for the systems and the camera placements.

#### 2.3.1. Optical Marker-Based System

A ten-camera passive marker Vicon^TM^ motion analysis system consisting of eight Bonita B10 and two Vero v1.3 cameras was used to acquire and pre-process motion data (VICON, Oxford, UK). The motion capture cameras were set to a sampling rate of 100 Hz. Prior to each data collection session, the system was calibrated according to manufacturer guidelines. Twenty-seven retro-reflective markers were placed on the upper body of each participant at bony anatomical landmarks of the upper body in accordance with the Vicon Upper-Body Plug-In-Gait body model documentation. The Plug-In-Gait upper body model was then calibrated to the dimensions of the participant to create the wrist, forearm, upper arm, head, neck, thorax, and pelvic model segments. The Vicon was set as the primary recording system and controlled the initiation and termination of IMU recordings with a voltage duration sync pulse output. 

#### 2.3.2. IMU System

Five IMUs for the Xsens Awinda were placed either at bony anatomical landmarks or the midpoints of moving body segments on the head, right arm, and torso. The head sensor was placed in the center of the subject’s forehead. The torso sensor was on the xiphoid process of the sternum. The pelvis sensor was placed at the midpoint between the left and right posterior superior iliac spines. The upper arm sensor was placed on the lateral midpoint of the upper arm. The forearm sensor was placed on the anterior midpoint of the bypass. The system was set to a sampling rate of 100 Hz. Prior to each data collection session, all accelerometry and gyroscope sensor outputs were set to zero at the origin of the recording volume on the floor, as defined by the Vicon calibration to ensure consistent initial sensor outputs. The Xsens was set as the secondary recording system with initiation and termination of recordings automatically controlled through a voltage duration sync pulse from the Vicon system, leading to the synchronization of two data streams from these systems.

#### 2.3.3. Markerless System

Two Kinect V1 cameras (Microsoft, Seattle, WA, USA) were used with the iPi Soft markerless motion capture software (iPi Soft, Moscow, Russia) to acquire and pre-process motion data. The Kinect V1 was selected due to the limitations of the native Microsoft SDK which did not allow for multiple Kinect V2 data streams into a single computer. This limitation did not apply to the Kinect V1, which allowed for larger capture volumes and improved capture results when multiple Kinect V1 sensors were used [74]. Additionally, the Azure Kinect was not commercially available and not supported by the iPi software at time of experiment. The Kinect cameras were positioned approximately ±45° from the midline of the subject at a distance of approximately 6 feet. The camera tripods were placed in the same position for each subject. The point of aim for the Kinect cameras was determined through the calibration procedures for the Kinect system and may vary depending on the experimental conditions.

The system was set to 30 Hz, the maximum sampling rate of the cameras. Prior to each data collection session, the system was calibrated according to the software manufacturer’s guidelines. Initiation and termination of recordings were manually controlled by the operator of the motion analysis systems. Data synchronization and resampling to 100 Hz with the built-in MATLAB function *resample* was achieved through a post-processing automated MATLAB script. This was done to create time series data that were sampled at the same rate to compare each distinct time point across systems. 

### 2.4. Data Analysis

Joint angles over time were generated for all three systems. The joint angle dataset from the Vicon system was set as the reference system given its high resolution and accuracy [1,2,3,4,5,6,7,8], as well as previous history of use as reference systems in research [27,36,37,38,44,45,48,65,66,67,68]. Root mean square error (RMSE) (1) and bias (2) were calculated for the IMU and markerless datasets. In Equations (1) and (2), *i* is the index for each frame in a given joint movement trajectory.

(1) $$\mathrm{RMSE}=\sqrt{\left(\frac{1}{n}\right)\sum_{i=1}^{n}{\left({\mathrm{testSystem}}_{i}-{\mathrm{Vicon}}_{i}\right)}^{2}},$$

(2) $$\mathrm{bias}=\left(\frac{1}{n}\right)\sum_{i=1}^{n}{\left({\mathrm{testSystem}}_{i}-{\mathrm{Vicon}}_{i}\right)}^{2},$$

A description of the joint angle calculations from each system used in the above equations is provided below.

To assess consistency of measurements from each system, the intraclass correlation coefficient (ICC) was calculated using a two-way mixed effects model (ICC(3,1)). Each participant performed three trials of the same task. These three trials were used to determine the ICC of discrete kinematic parameters derived from the joint trajectory (range of motion) for each task/DoF combination and for each motion system evaluated in our study. The use of discrete kinematic parameters, such as RoM, was used to avoid artificially low ICC values due to slight misalignments in the trajectories across trials within a subject. 

#### 2.4.1. Optical Marker-Based System

Joint angles were calculated from the Vicon upper body model using YXZ Euler angles derived from relative orientation comparisons of two segments (VICON Plug-in Gait, Oxford, UK). Joint angles analyzed in this study include right elbow flexion/extension; right shoulder flexion/extension, abduction/adduction, and internal/external rotation; torso flexion, lateral flexion, and rotation; and neck flexion, lateral flexion, and rotation. 

The recorded data from each task were processed and manually segmented in Vicon Nexus into object interactions with the beginning of a segment defined as when the terminal device approached the object, and the end of a segment when the terminal device released the object. For tasks with multiple objects, such as the six objects in JHFT—Small Objects, this resulted in multiple segments. Although the locations of the task objects are standardized with placement templates, the individual objects may be distributed in the task space. Therefore, to reduce variability introduced in joint kinematics due to object distribution, the analysis was limited to the last segment, or last object interaction, within each trial.

#### 2.4.2. IMU System

The joint angles for the IMU-based system were calculated based on relative sensor orientation. To generate joint angles, the IMUS proximal and distal to each joint were paired: one sensor was defined as the parent sensor and used to establish a local coordinate system and the other sensor was defined as the child sensor and provided the orientation data necessary to generate the joint angles. The right elbow flexion/extension was calculated between the forearm and upper arm sensors. Right shoulder angles were calculated between the sternum and upper arm sensors. Neck angles were calculated from the head and sternum sensors. Torso angles were calculated between the sternum and pelvis sensors. A brief description of the IMU joint angle calculation process is described below, with further details in Appendix A.

The IMU sensor orientations were output as quaternions and decomposed into axial vector components that corresponded to the three axes of the sensor units in unit quaternions. Then, the axial vector components were used to generate the individual Euler joint angle components through decomposition. The decomposition used the known initial orientations of the sensor unit locations on the body to define a superior-inferior axis for each sensor, with the other two axes defined through orthogonality. The angles defined through the pairs of sensors sought to mimic the output of the Vicon YXZ Euler angle outputs. However, the shoulder angles suffered from computational errors and the XYZ rotation order was used instead to best match the Vicon outputs. This approach matched what was found in a recent study [32]. The planar surface sensor calibrations and known body placement locations were used for the alignment of sensor axes to body segment axes. The initial values of the Vicon outputs were used to initialize the values of the derived Xsens angles to limit the variance from the calibration approach. In some instances, joint angles from the Xsens IMU system were inverted to match the conventions of the Vicon reference system angle values. The resultant angles were then visually examined for computational anomalies that violated anatomical angle limits due to gimble lock. Trajectories with computational anomalies were manually removed from the analysis.

Since the data from the optical marker-based and IMU systems were synchronized, the IMU data were segmented for analysis using the segmentation event markers from the optical marker-based system. As previously mentioned, analysis was limited to the last segment of each task.

#### 2.4.3. Markerless System

The joint angles for the dual Kinects were calculated with the Biomech add-on toolbox for iPi studio using YXZ Euler angles derived from the relative orientation comparisons of two skeletal rig segments (iPi Soft, Moscow, Russia). To derive joint angles comparable to those generated from the Vicon marker-based system, re-zeroing operations were performed on the outputs of the Biomech toolbox. In some instances, joint angles from the Kinect system were inverted to match the conventions of the Vicon reference system angle values. For the right elbow angles, due to the obscuration of the MYO Bypass device caused by the actual right arm, the Kinect prioritized arm tracking over bypass tracking. An offset of 15°, equal to the medial offset of the bypass device, was applied to these elbow angles to provide a more accurate estimate. 

The data from the markerless system were synced with the optical marker-based and IMU systems post-capture with an automated MATLAB script. To aid in this synchronization, all subjects started each trial with their hands at their side and subsequently moved their arms into a “motor-bike” pose before performing the task. The transition to the motor-bike pose caused a predictable spike in the right shoulder angle. The MATLAB script detected time points in each system where the joint angle rate of change, or joint angle derivative, in the right shoulder exceeded a preset threshold (determined through pilot experiments). The data from each system were aligned to this detected time point and the first data point of the markerless system was adjusted to match the Vicon data point to be consistent. As previously mentioned, analysis was limited to the last segment of each task.

## 3. Results

**Figure 2:**
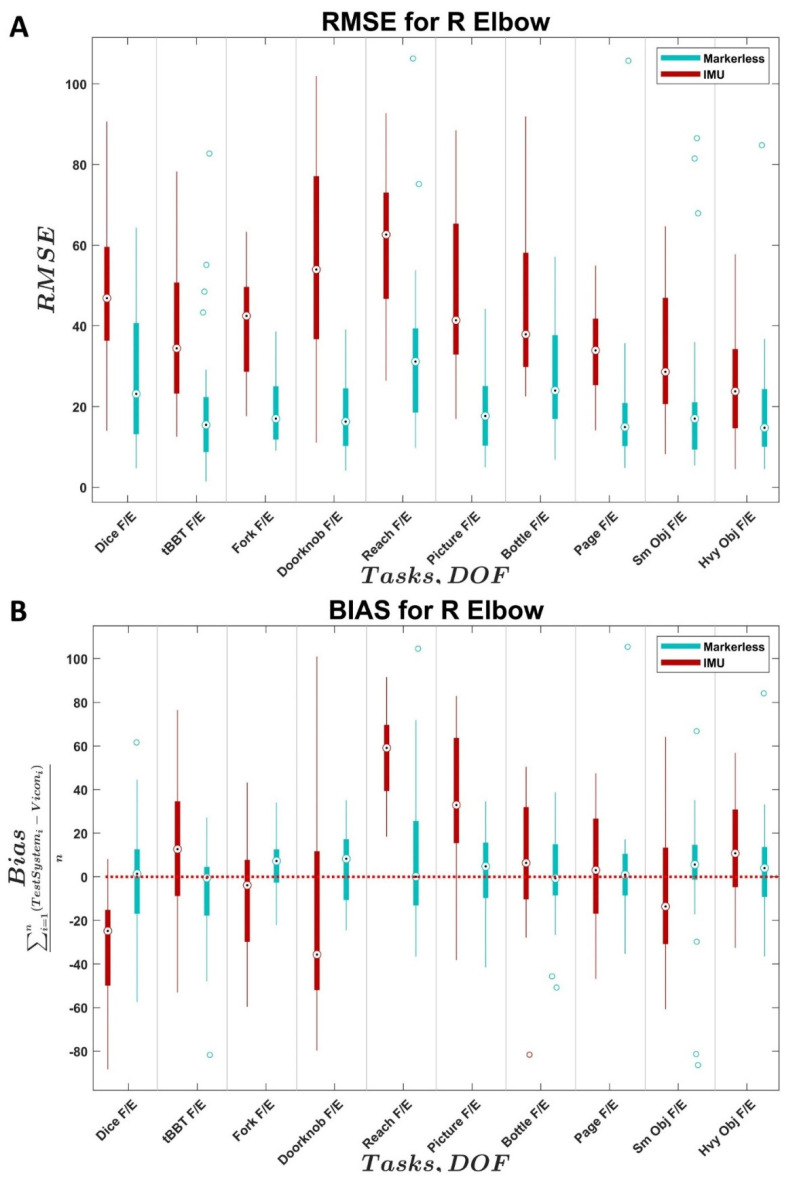
Distributions of (**A**) right elbow flexion RMSE and (**B**) right elbow flexion bias across subjects for the IMU system (Xsens Awinda MTw, El Segundo, CA) and markerless system (Kinect, Microsoft, Seattle, WA, USA) compared to the reference system (VICON, Oxford, UK). *X*-axis identifies the task and associated joint angle. F/E = flexion/extension, Ab/Ad = abduction/adduction, LaF = lateral flexion, Rot = rotation. Black dots indicate medians, empty circles indicate outliers, bold line indicates quartiles, and whiskers indicate non-outlier maximums and minimum.

**Figure 3:**
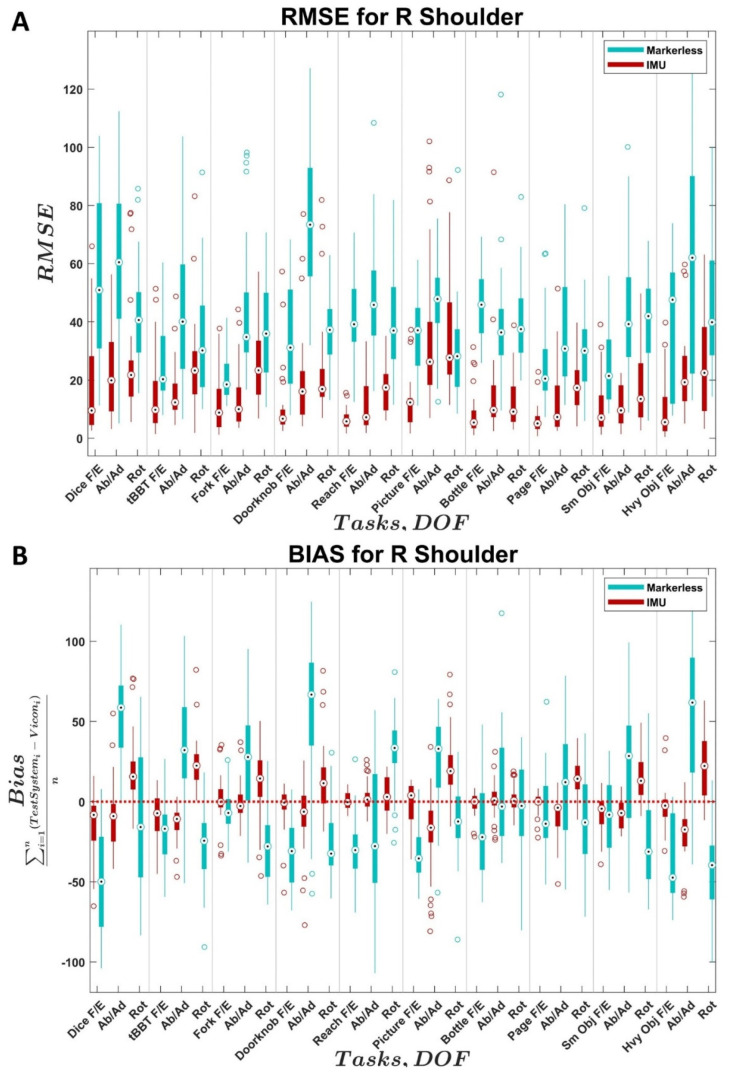
Distributions of (**A**) right shoulder joint angle RMSE and (**B**) right shoulder joint angle bias across subjects for the IMU system (Xsens Awinda MTw, El Segundo, CA, USA) and markerless system (Kinect, Microsoft, Seattle, WA, USA) compared to the reference system (VICON, Oxford, UK). *X*-axis identifies the task and associated joint angle. F/E = flexion/extension, Ab/Ad = abduction/adduction, LaF = lateral flexion, Rot = rotation. Black dots indicate medians, empty circles indicate outliers, bold line indicates quartiles, and whiskers indicate non-outlier maximums and minimum.

**Figure 4:**
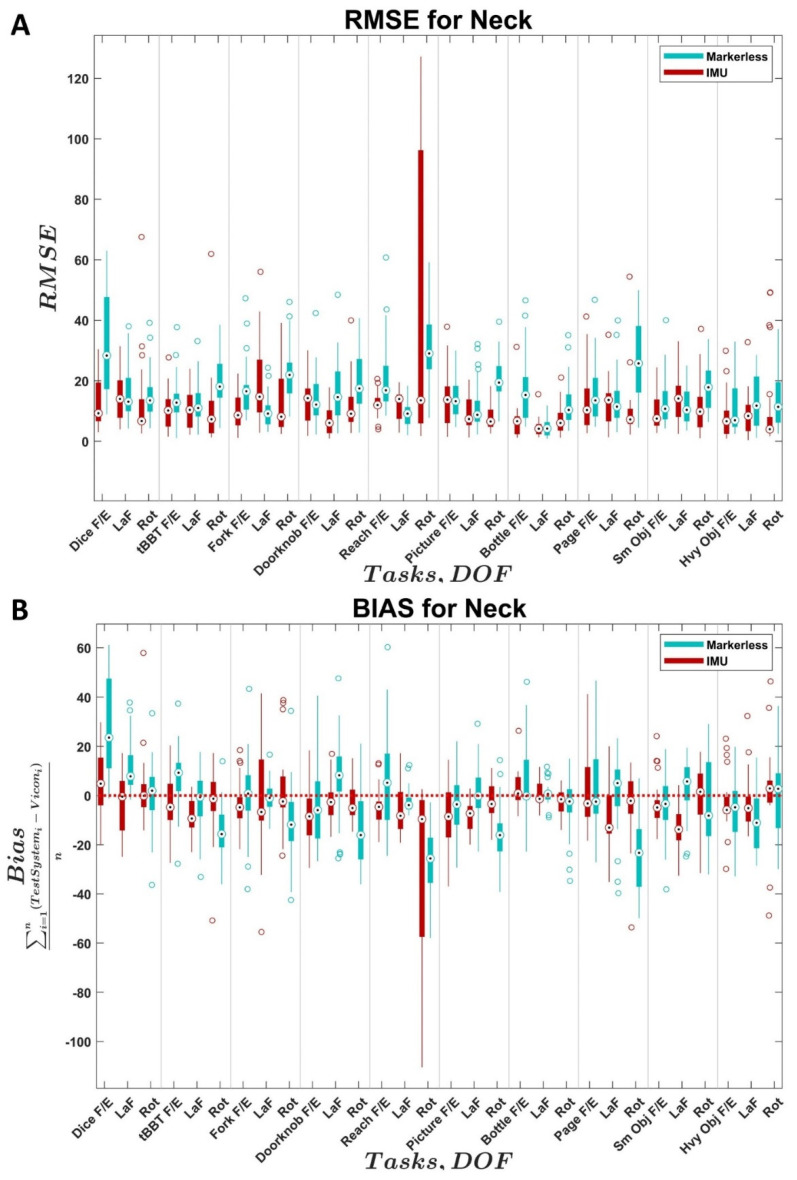
Distributions of (**A**) neck joint angle RMSE and (**B**) neck joint angle bias across subjects for the IMU system (Xsens Awinda MTw, El Segundo, CA) and markerless system (Kinect, Microsoft, Seattle, WA, USA) compared to the reference system (VICON, Oxford, UK). *X*-axis identifies the task and associated joint angle. F/E = flexion/extension, Ab/Ad = abduction/adduction, LaF = lateral flexion, Rot = rotation. Black dots indicate medians, empty circles indicate outliers, bold line indicates quartiles, and whiskers indicate non-outlier maximums and minimum.

**Figure 5:**
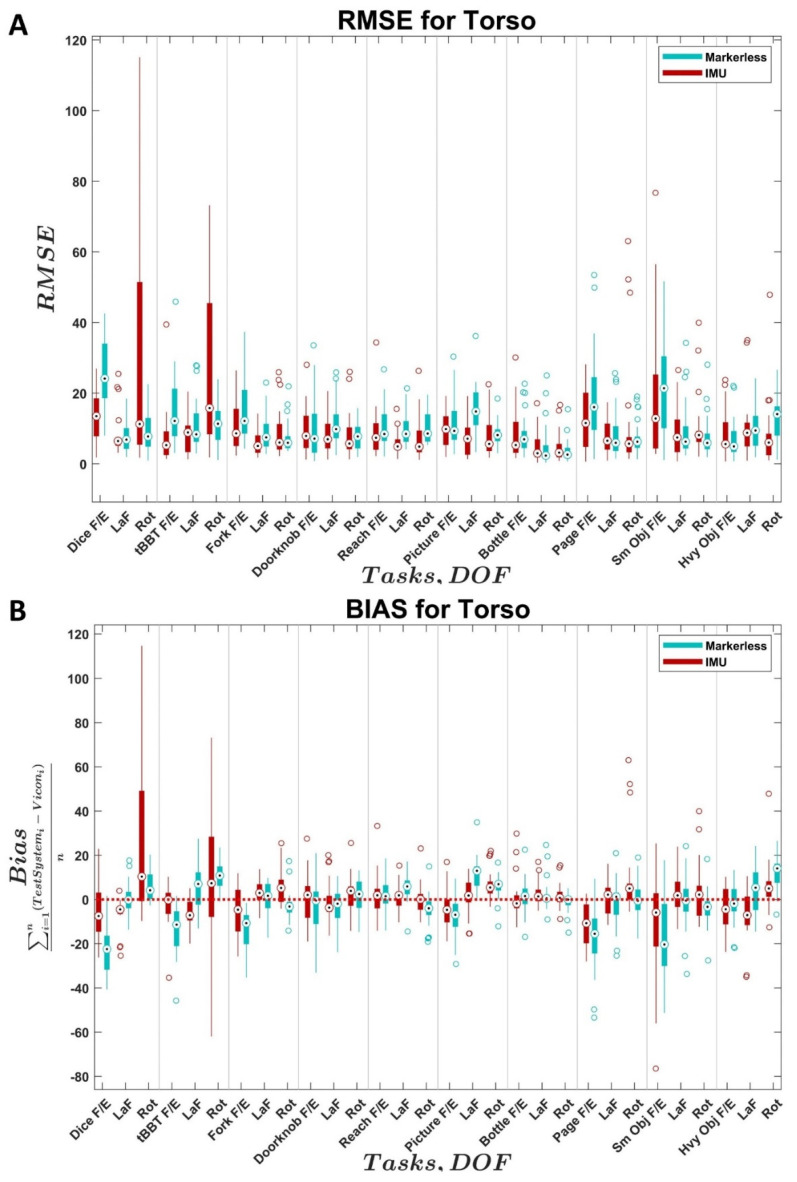
Distributions of (**A**) torso angle RMSE and (**B**) torso angle bias across subjects for the IMU system (Xsens Awinda MTw, El Segundo, CA) and markerless system (Kinect, Microsoft, Seattle, WA, USA) compared to the reference system (VICON, Oxford, UK). *X*-axis identifies the task and associated joint angle. F/E = flexion/extension, Ab/Ad = abduction/adduction, LaF = lateral flexion, Rot = rotation. Black dots indicate medians, empty circles indicate outliers, bold line indicates quartiles, and whiskers indicate non-outlier maximums and minimum.

The distributions of RMSE and bias values across all trials and subjects for the two comparison systems relative to the reference system are shown as boxplots for each joint in Figure 2, Figure 3, Figure 4 and Figure 5. In each figure, the values for the IMU system are shown in red; values for the markerless system are shown in blue. Within each distribution, white circles with a black dot indicate the median of the distribution. 

The markerless system tended to slightly overestimate the right elbow angle while the IMU system was inconsistent and greater in magnitude in the bias measurement (Figure 2B). Larger errors were seen with the IMU system for right elbow flexion: the median RMSE values for the markerless system were between 14.4° and 31.2° while the median RMSE values for the IMU system were between 23.8° and 62.6°. The AMULA—Reach task had the highest median RMSE values across both systems (Figure 2A). This task resulted in a relatively low bias value across the tasks for the markerless system at 8.4°, and the most positive bias value for the IMU system at 59.1°. 

Conversely, with the right shoulder, the IMU system had lower median RMSE values and tended to have lower variance for RMSE and bias compared to the markerless system (Figure 3A,B). The median RMSE values across tasks and DOFs for the IMU system were all under 30° while median RMSE values at the shoulder for the markerless system were above 30°. The markerless system tended to underestimate shoulder flexion/extension and shoulder rotation while overestimating shoulder adduction/abduction. In contrast, the IMU system tended to overestimate shoulder rotation and underestimate shoulder abduction/adduction across tasks. Compared to the markerless system, the median bias values for the IMU system tended to be closer to zero across all tasks and DOFs (Figure 3B). The tasks with the lowest RMSE values and the bias values closest to zero varied depending on the joint angle component. For the IMU system, JHFT—Page Turn had the lowest median RMSE for shoulder flexion/extension at 5.1° and the task’s median bias was the closest to zero for all tasks in the shoulder flexion/extension component at 0.02°. The AMULA—Reach task had the lowest median IMU RMSE for shoulder abduction/adduction at 7.2°, and the corresponding median bias was 1.3°. CAPPFUL—Bottle had the lowest median IMU RMSE for shoulder rotation at 9.2°. For the markerless system: the lowest median shoulder flexion/extension RMSE value was in the AMULA—Fork task (18.5°), the lowest median shoulder abduction/adduction RMSE value was in the JHFT—Page task (30.8°), and the smallest median shoulder rotation RMSE value was in the CAPPFUL—Picture task (28.2°). The bias values closest to zero for all tasks in the markerless system were in: AMULA—Fork for shoulder flexion/extension (−7.0°), CAPPFUL—Bottle for shoulder abduction/adduction (−3.1°), and CAPPFUL—Bottle for shoulder rotation (−2.6°).

With the neck angle measurements (Figure 4A,B), the IMU system tended to have slightly lower RMSE values and comparable variance compared to the markerless system. For the IMU system, neck rotation in the AMULA—Reach was a notable outlier in the variance even though the median RMSE value of 13.9° was in line with the magnitude of the neck rotation values found in other tasks. Similarly, the markerless system had the largest median RMSE value in AMULA—Reach neck rotation at 29.04°. The IMU system was more closely clustered around zero for the bias values compared to the markerless system. The median RMSE and bias values that were closest to zero were distributed across the JHFT—Heavy Objects, CAPPFUL—Bottle, and CAPPFUL—Dice tasks for the three components of the neck across the two systems. In the IMU system, the median RMSE values ranged from 6.6° to 14.7° while bias values ranged from −13.7° to 4.9°; for the markerless system, the median RMSE values ranged from 4.2° to 28.3° and the median bias values ranged from −25.6° to 23.6°.

With the torso angle measurements (Figure 5A,B), the IMU system tended to have slightly lower median RMSE values compared to the markerless system. However, the IMU system had much greater variance in torso rotation RMSE values in the CAPPFUL—Dice and tBBT tasks. The markerless system had the greatest median RMSE values and greatest RMSE variance in torso flexion for the JHFT—Page Turn and JHFT—Small Objects tasks. For both systems, the task with the lowest median RMSE values for all torso components was CAPPFUL—Bottle. For the IMU, the torso flexion/extension was 5.30°, the torso lateral flexion was 2.9°, and the torso rotation was 3.2°. For the markerless system, the torso flexion/extension was 6.9°, the torso lateral flexion was 2.3°, and the torso rotation was 2.6°. With the IMU system, the median RMSE values ranged from 3.2° to 15.8° and the bias values ranged from −10.7° to 10.3°; with the markerless system, the median RMSE values ranged from 2.3° to 24.1° and the bias values ranged from −22.5° to 14.0°.

Table 2 shows the ICC(3,1) along with the 95% confidence interval for each system, DOF, and task combination. ICC values less than 0.4 were considered weak correlation; values between 0.4 and 0.74 were considered moderate, and values equal to or greater than 0.75 were considered strong [75]. To facilitate the qualitatively comparison of ICC across systems, the table is color-coded according to the weak, moderate, and strong definitions. In general, the Vicon and IMU systems have moderate to strong correlations across trials for all subjects. There does not appear to be any trend based on the task or DoF. The Kinect system generally has poor reliability with weaker ICC values. 

## 4. Discussion

In this study, joint kinematics derived from three motion capture systems of varying costs and mechanisms were compared through simultaneous motion capture of able-bodied participants using an upper limb myoelectric bypass device. By evaluating the ability of each system to capture kinematic changes of simulated upper limb prosthesis users during a variety of standardized tasks, this study provides insight into the advantages and limitations of using different motion capture technologies for upper limb functional assessment. Two established metrics of precision and accuracy (RMSE and bias) were calculated as a function of ten different joint degrees of freedom and ten different upper-limb tasks for every time point to assess inter-subject variability and inter-system agreement. Because differences are calculated for every time point, the RMSE values would reflect unstable system-related influences given the simultaneous capture setup. Similarly, the bias values would indicate systematic influences on differences over time—allowing for an assessment of joint angle stability. In addition, ICC values were calculated for each system and each task/DoF combination using a two-way mixed effects model (ICC(3,1) to further assess consistency of measurements from each system. A discussion of advantages and limitations of each system is presented along with considerations for clinical implementation. 

Based on the results presented in this study, the IMU system yields more accurate kinematics for shoulder, neck, and torso angles over all DOFS (Figure 3, Figure 4 and Figure 5) compared to the Kinect (markerless) system’s performance over all DOFs. Due to the current level of accuracy and variability, the IMU system is not recommended in the elbow DOF (Figure 2). The markerless system is not recommended for use in measuring the elbow or the shoulder DOFs due to high variability and bias (Figure 2 and Figure 3), which are in line with the results from the literature [23,27,35], but may provide accurate results for neck and torso DOFs (Figure 4 and Figure 5) when individuals perform the specific tasks analyzed in this study. 

For both systems, the tasks requiring the greatest amount of movement (i.e., CAPPFUL—Dice, CAPPFUL—Picture, and tBBT) resulted in the largest RMSE and variability values over the DOFs examined. This implies that both systems struggled with precision during large gross movements, a result in line with the previous literature that suggests the markerless system overestimates large motions and underestimates small motions [27].

For DOFs parallel to the recording plane of the cameras (e.g., neck/torso lateral flexion and shoulder abduction/adduction), the markerless system had the best results. Given the mechanism of movement capture for the Kinect V1, which measures infrared reflectivity and subtracts changes from a predefined static background [24,46,73], this result is expected [38,45,73]. The elbow bias values (Figure 2B) for the markerless system were inconsistent overestimates, which were likely influenced by how the system struggled to detect the bypass device. The markerless shoulder bias values (Figure 3B) measured in this study were also notably different from those found in the literature (current study measured approximately −25° compared to an average around +10°) [26,27]. Although this difference is large, it may be a more accurate representation of the expected performance of these motion capture systems given the use of complex tasks [76] and human subjects in this study compared to simple ROM measurements [26,27] and testing machines [47] found in the literature. 

The precision of the IMU system was best in the shoulder (Figure 3), which is consistent with previous results in the literature [28,32]. The variability across subjects in the elbow DOF (Figure 2) for the IMU system was likely influenced by variations in sensor placement and movement artifacts from the sensor attachment method, which are known factors in the literature [39]. The variability across subjects in the neck and torso angles (Figure 4 and Figure 5) appeared to be heavily task-influenced and the capture accuracy of the systems was likely affected by the varying motions used by the participants to achieve the task. The magnitude of the differences between the IMU-generated angles and the Vicon reference system angles found in the DOFs examined in this study were similar to the magnitude of the differences previously found in the literature for the commercial Xsens software [76] in the shoulder, neck, and back. However, the magnitude of the differences in the elbow are much greater in this study compared to those previously found in the literature. The source of the errors within the elbow is currently still unclear and warrants further investigation given the results seen in the other angles measured.

In terms of the system stability as measured by the ICC values, the IMU and marker-based systems showed comparable moderate to strong correlations across trials for all subjects. The markerless system generally showed weaker correlations compared to the IMU system and marker-based reference system. Due to the lack of any trends based on the task or DoF, these results can be considered to support the general performance of the three systems. However, it should be noted that the participants were free to choose their own approaches to achieve the tasks and often used different approaches between trials. As such, it is difficult to draw more specific conclusions based on the ICC values due to the inherent variability of the base data. Overall, the marker-based reference system and IMU system showed the greatest stability per the ICC metric.

In terms of capturing environment restrictions and operating stability, the IMU system proved more robust and less demanding compared to the markerless system. The IMU system did not require the consideration of issues such as the color and reflectivity of the capture background and was not vulnerable to issues of obscuration from task objects or body parts. The IMU system had comparable costs for the number of sensors used, and less strenuous requirements for data processing, data storage, and data export procedures compared to the markerless system. The markerless system incorporated established calibration procedures, while there exist many approaches for effective calibration of the IMU system. The impact of IMU calibration procedure on derived joint angles was not the subject of this study but may need further investigation regarding the most effective calibration approach. However, both systems proved lacking in data annotation abilities—with the markerless system holding a slight advantage due to the visual review allowed by the video-based capture data. Overall, the IMU system may be best for clinical and remote monitoring purposes.

The generalizability of the joint kinematics observed here with able-bodied individuals to those of upper limb prosthesis users is uncertain. However, the movements elicited by able-bodied individuals using a bypass prosthesis are close approximations to the movements of interest, making the results obtained in this study relevant to understanding the utility of different motion capture systems for tracking upper limb prosthesis user movement. The limitations and advantages discovered about each system in this study can be used to inform clinical implementation of motion analysis for research and rehabilitation. The focus on unilateral tasks performed with the MYO Bypass device may not be fully representative of device use patterns in daily living and may also be considered a limitation of the current study. While a bilateral task was included, (i.e., CAPPFUL Task 11—Picture), the task required symmetrical use of the two upper limbs. Motion analysis of tasks with independent use of both upper limbs has yet to be performed under these simultaneous capture conditions and is a future avenue of investigation. Given the current results with unilateral tasks, and other results in the literature [41], it is likely that asymmetrical bilateral tasks may further elucidate the performance capabilities of the IMU and the markerless motion analysis systems. Future work may also focus on investigating the effects of additional Kinect V1 cameras, the results from more modern Kinect cameras models such as the Azure Kinect, and refinement of the IMU system joint angle calculations and sensor placements to allow for more reliable capture of challenging task performance zones such as the portion of the lower central zone by the feet and the far left and far right of the lateral zones [49]. Future work may also include remote monitoring and additional capture mechanisms such as those employed in visual-inertial systems or single-view pose estimation systems. 

## 5. Conclusions

This study is the first that simultaneously compares multiple mechanisms of motion capture using a simulated upper limb prosthesis user population. It can serve as a starting point for minimum technical requirements in motion capture systems for use in clinical rehabilitation and highlights the current state of commercially available technology in terms of technical and capture environment requirements that may be barriers to the clinical adoption of motion capture. The results from this study can also be used to guide improvements in the design and algorithms of low-cost, portable motion capture systems to facilitate the wider adoption of these tools in clinical practice.

## Figures and Tables

**Table 1:** Description of tBBT and tasks from the JHFT, AMULA, and CAPPFUL performed by study participants.

OM—Task Name	Description
JHFT—Page Turn	Flip over five 3 × 5-inch notecards arranged in a row with any technique, starting with the leftmost card and moving across.
JHFT—Small Objects	Pick up six small objects (2 paperclips, 2 bottle caps, and 2 pennies) arranged two inches apart on the dominant side of the subject, and place in an empty can individually, starting with the right most object.
JHFT—Heavy Objects	Lift 5 filled cans individually about 1 inch onto a board, starting with the rightmost can.
AMULA—Fork	Grasp fork and bring to mouth, move fork back to table and release fork.
AMULA—Doorknob	Reach, grasp, and turn doorknob. Release doorknob.
AMULA—Reach	Lift arm overhead to grasp empty cup on shelf and bring down arm with cup in hand.
CAPPFUL—Dice	Pick up three dice from a plate, touch to chin, and return to plate.
CAPPFUL—Bottle	Empty a squeeze bottle of water into a cup.
CAPPFUL—Picture	Modified task—Reach overhead to grasp two rings suspended in the air on a pulley system, bring rings down to waist, then control the placement of rings back in their suspended position.
tBBT	Transport 16 blocks, one at a time, over a partition using only the dominant hand, starting with the innermost left block and moving across each row placing the block in its mirrored position.

**Table 2:** ICC values with 95% confidence intervals for each DoF, task, and motion system comparison. Red cells indicate a weak correlation (ICC < 0.4). Yellow cells indicate a moderate correlation (0.4 ≤ ICC < 0.75). Green cells indicate a strong correlation (ICC ≥ 0.75).

Joint/DoF	Tasks	ICC (Kinect)	95% CI	ICC (Vicon)	95% CI	ICC (Xsens)	95% CI
**Right Elbow F/E**	CAPPFUL4	0.73	[0.42, 0.92]	0.88	[0.6, 0.98]	0.88	[0.59, 0.98]
	tBBT	0.21	[−0.15, 0.66]	0.76	[0.32, 0.96]	0.66	[0.17, 0.94]
	AMULA10	0.31	[−0.071, 0.72]	0.72	[0.34, 0.93]	0.91	[0.75, 0.98]
	AMULA16	0.19	[−0.18, 0.66]	0.47	[0.025, 0.84]	0.85	[0.59, 0.96]
	AMULA24	0.86	[0.64, 0.96]	0.68	[0.2, 0.94]	0.80	[0.42, 0.97]
	CAPPFUL11	0.69	[0.35, 0.9]	0.36	[−0.073, 0.79]	0.79	[0.46, 0.95]
	CAPPFUL8	0.47	[0.077, 0.81]	0.95	[0.85, 0.99]	0.82	[0.53, 0.96]
	JHFT2	0.33	[−0.05, 0.73]	−0.16	[−0.38, 0.36]	0.18	[−0.21, 0.69]
	JHFT3	0.19	[−0.16, 0.64]	0.54	[0.096, 0.87]	0.41	[−0.029, 0.81]
	JHFT7	0.40	[−0.014, 0.79]	0.63	[0.21, 0.9]	0.69	[0.3, 0.92]
**Right Shoulder F/E**	CAPPFUL4	0.66	[0.31, 0.89]	0.92	[0.75, 0.98]	0.91	[0.73, 0.98]
	tBBT	0.22	[−0.14, 0.66]	0.56	[0.12, 0.87]	0.57	[0.14, 0.88]
	AMULA10	0.50	[0.1, 0.82]	0.65	[0.24, 0.91]	0.69	[0.3, 0.92]
	AMULA16	0.33	[−0.077, 0.75]	0.73	[0.4, 0.92]	0.58	[0.18, 0.87]
	AMULA24	0.73	[0.4, 0.92]	0.88	[0.7, 0.97]	0.92	[0.79, 0.98]
	CAPPFUL11	0.42	[0.024, 0.78]	0.87	[0.68, 0.96]	0.80	[0.54, 0.94]
	CAPPFUL8	0.16	[−0.19, 0.61]	0.98	[0.93, 0.99]	0.78	[0.49, 0.93]
	JHFT2	−0.12	[−0.34, 0.33]	0.67	[0.3, 0.9]	0.66	[0.29, 0.9]
	JHFT3	0.66	[0.31, 0.89]	0.78	[0.48, 0.94]	0.75	[0.43, 0.93]
	JHFT7	0.23	[−0.15, 0.69]	0.80	[0.5, 0.95]	0.81	[0.52, 0.96]
**Right Shoulder Ab/Ad**	CAPPFUL4	0.47	[0.072, 0.8]	0.66	[0.25, 0.91]	0.63	[0.21, 0.9]
	tBBT	0.10	[−0.22, 0.57]	0.42	[−0.02, 0.82]	0.64	[0.22, 0.9]
	AMULA10	0.38	[−0.014, 0.76]	0.71	[0.33, 0.93]	0.90	[0.7, 0.98]
	AMULA16	0.50	[0.081, 0.83]	0.76	[0.44, 0.93]	0.51	[0.094, 0.84]
	AMULA24	0.83	[0.57, 0.95]	0.90	[0.74, 0.97]	0.91	[0.77, 0.97]
	CAPPFUL11	0.46	[0.065, 0.8]	0.85	[0.63, 0.96]	0.89	[0.72, 0.97]
	CAPPFUL8	0.51	[0.11, 0.82]	0.91	[0.76, 0.97]	0.90	[0.75, 0.97]
	JHFT2	0.37	[−0.021, 0.75]	0.69	[0.33, 0.91]	0.79	[0.49, 0.94]
	JHFT3	0.39	[0.0016, 0.77]	0.79	[0.49, 0.94]	0.86	[0.64, 0.96]
	JHFT7	−0.01	[−0.3, 0.5]	0.50	[0.062, 0.85]	0.42	[−0.017, 0.82]
**Right Shoulder Rot**	CAPPFUL4	0.78	[0.49, 0.93]	0.92	[0.75, 0.98]	0.79	[0.47, 0.95]
	tBBT	0.11	[−0.21, 0.58]	0.61	[0.18, 0.89]	0.60	[0.17, 0.89]
	AMULA10	0.44	[0.048, 0.79]	0.84	[0.56, 0.96]	0.97	[0.91, 0.99]
	AMULA16	0.15	[−0.2, 0.64]	0.77	[0.46, 0.94]	0.51	[0.099, 0.84]
	AMULA24	0.44	[0.022, 0.81]	0.92	[0.78, 0.98]	0.89	[0.71, 0.97]
	CAPPFUL11	0.49	[0.096, 0.82]	0.73	[0.42, 0.92]	0.79	[0.52, 0.94]
	CAPPFUL8	0.84	[0.61, 0.95]	0.56	[0.17, 0.85]	0.70	[0.36, 0.9]
	JHFT2	0.78	[0.49, 0.93]	0.18	[−0.19, 0.65]	0.56	[0.15, 0.86]
	JHFT3	0.35	[−0.033, 0.75]	0.66	[0.28, 0.9]	0.78	[0.48, 0.94]
	JHFT7	−0.11	[−0.35, 0.38]	0.53	[0.089, 0.86]	0.45	[0.0077, 0.83]
**Neck F/E**	CAPPFUL4	0.54	[0.15, 0.84]	0.52	[0.0014, 0.9]	−0.10	[−0.38, 0.56]
	tBBT	−0.03	[−0.3, 0.45]	0.17	[−0.26, 0.76]	0.05	[−0.32, 0.69]
	AMULA10	0.30	[−0.082, 0.71]	0.60	[0.14, 0.91]	0.60	[0.14, 0.91]
	AMULA16	0.55	[0.14, 0.86]	0.28	[−0.14, 0.75]	0.42	[−0.019, 0.82]
	AMULA24	0.52	[0.11, 0.84]	0.96	[0.9, 0.99]	0.97	[0.91, 0.99]
	CAPPFUL11	0.31	[−0.075, 0.72]	0.54	[0.13, 0.85]	0.86	[0.63, 0.96]
	CAPPFUL8	0.72	[0.4, 0.91]	0.76	[0.38, 0.95]	0.58	[0.11, 0.9]
	JHFT2	0.31	[−0.089, 0.74]	0.69	[0.31, 0.92]	0.20	[−0.19, 0.7]
	JHFT3	0.29	[−0.084, 0.71]	0.68	[0.19, 0.94]	0.36	[−0.14, 0.85]
	JHFT7	−0.02	[−0.3, 0.49]	0.76	[0.41, 0.94]	0.86	[0.62, 0.97]
**Neck LaF**	CAPPFUL4	−0.23	[−0.39, 0.17]	0.75	[0.3, 0.96]	0.86	[0.54, 0.98]
	tBBT	0.23	[−0.13, 0.67]	0.49	[−0.029, 0.89]	0.37	[−0.13, 0.85]
	AMULA10	0.59	[0.22, 0.86]	0.64	[0.18, 0.92]	0.79	[0.43, 0.96]
	AMULA16	0.26	[−0.13, 0.71]	0.23	[−0.17, 0.72]	−0.12	[−0.36, 0.41]
	AMULA24	0.69	[0.33, 0.91]	0.93	[0.81, 0.98]	0.93	[0.79, 0.98]
	CAPPFUL11	0.44	[0.048, 0.79]	0.85	[0.62, 0.96]	0.90	[0.74, 0.98]
	CAPPFUL8	0.62	[0.25, 0.87]	0.74	[0.34, 0.94]	0.67	[0.23, 0.92]
	JHFT2	0.11	[−0.23, 0.61]	0.66	[0.25, 0.91]	0.63	[0.21, 0.9]
	JHFT3	0.48	[0.087, 0.81]	0.85	[0.53, 0.98]	0.92	[0.71, 0.99]
	JHFT7	0.09	[−0.24, 0.58]	0.39	[−0.048, 0.81]	0.48	[0.034, 0.84]
**Neck Rot**	CAPPFUL4	−0.05	[−0.31, 0.42]	0.91	[0.69, 0.99]	0.13	[−0.28, 0.74]
	tBBT	0.13	[−0.2, 0.59]	0.71	[0.25, 0.95]	0.47	[−0.051, 0.88]
	AMULA10	0.60	[0.23, 0.87]	0.89	[0.66, 0.98]	0.56	[0.09, 0.89]
	AMULA16	0.25	[−0.13, 0.71]	0.31	[−0.12, 0.76]	0.38	[−0.057, 0.8]
	AMULA24	0.59	[0.19, 0.87]	0.93	[0.8, 0.98]	0.99	[0.98, 1]
	CAPPFUL11	0.54	[0.16, 0.84]	0.89	[0.7, 0.97]	0.80	[0.52, 0.95]
	CAPPFUL8	0.51	[0.12, 0.82]	0.60	[0.14, 0.9]	0.71	[0.29, 0.94]
	JHFT2	0.27	[−0.12, 0.71]	0.60	[0.18, 0.89]	0.15	[−0.22, 0.67]
	JHFT3	0.36	[−0.028, 0.75]	0.91	[0.68, 0.99]	0.69	[0.21, 0.94]
	JHFT7	0.09	[−0.24, 0.59]	0.74	[0.37, 0.93]	0.68	[0.28, 0.92]
**Torso F/E**	CAPPFUL4	0.82	[0.58, 0.95]	0.98	[0.93, 1]	0.90	[0.65, 0.98]
	tBBT	−0.07	[−0.32, 0.4]	0.52	[0.043, 0.88]	0.58	[0.12, 0.9]
	AMULA10	0.50	[0.11, 0.82]	0.70	[0.27, 0.93]	0.69	[0.26, 0.93]
	AMULA16	0.06	[−0.26, 0.56]	0.59	[0.22, 0.86]	0.42	[0.032, 0.78]
	AMULA24	0.07	[−0.25, 0.57]	0.96	[0.87, 0.99]	0.93	[0.79, 0.98]
	CAPPFUL11	0.46	[0.066, 0.8]	0.90	[0.74, 0.97]	0.60	[0.23, 0.87]
	CAPPFUL8	0.62	[0.25, 0.87]	0.83	[0.57, 0.95]	0.68	[0.31, 0.91]
	JHFT2	0.40	[0.0059, 0.77]	0.48	[0.083, 0.81]	0.72	[0.4, 0.91]
	JHFT3	0.34	[−0.044, 0.74]	0.89	[0.73, 0.97]	0.90	[0.73, 0.97]
	JHFT7	0.70	[0.35, 0.91]	0.57	[0.16, 0.86]	0.39	[−0.023, 0.78]
**Torso LaF**	CAPPFUL4	0.67	[0.32, 0.89]	0.66	[0.17, 0.94]	0.78	[0.36, 0.96]
	tBBT	0.27	[−0.1, 0.7]	0.71	[0.29, 0.94]	0.66	[0.22, 0.92]
	AMULA10	0.72	[0.4, 0.91]	0.82	[0.49, 0.96]	0.35	[−0.11, 0.81]
	AMULA16	0.19	[−0.18, 0.67]	0.90	[0.74, 0.97]	0.72	[0.4, 0.91]
	AMULA24	0.11	[−0.23, 0.6]	0.90	[0.73, 0.97]	0.89	[0.7, 0.97]
	CAPPFUL11	0.55	[0.16, 0.84]	0.88	[0.7, 0.97]	0.95	[0.87, 0.99]
	CAPPFUL8	0.25	[−0.11, 0.68]	0.90	[0.73, 0.97]	0.94	[0.82, 0.98]
	JHFT2	0.28	[−0.093, 0.7]	0.61	[0.23, 0.87]	0.88	[0.69, 0.96]
	JHFT3	0.40	[0.01, 0.77]	0.57	[0.19, 0.85]	0.88	[0.69, 0.96]
	JHFT7	0.38	[−0.028, 0.78]	0.70	[0.34, 0.91]	0.33	[−0.076, 0.75]
**Torso Rot**	CAPPFUL4	0.46	[0.063, 0.8]	0.40	[−0.11, 0.86]	0.50	[−0.023, 0.89]
	tBBT	0.27	[−0.1, 0.69]	0.73	[0.32, 0.94]	0.62	[0.17, 0.91]
	AMULA10	0.69	[0.35, 0.9]	0.80	[0.45, 0.96]	0.85	[0.57, 0.97]
	AMULA16	0.36	[−0.046, 0.77]	0.63	[0.27, 0.88]	0.54	[0.15, 0.84]
	AMULA24	−0.08	[−0.34, 0.42]	0.89	[0.7, 0.97]	0.96	[0.89, 0.99]
	CAPPFUL11	0.62	[0.26, 0.88]	0.62	[0.25, 0.87]	0.67	[0.33, 0.9]
	CAPPFUL8	0.49	[0.094, 0.82]	0.93	[0.81, 0.98]	0.89	[0.7, 0.97]
	JHFT2	0.06	[−0.25, 0.53]	0.68	[0.33, 0.9]	0.53	[0.14, 0.83]
	JHFT3	0.16	[−0.18, 0.61]	0.85	[0.63, 0.96]	0.63	[0.27, 0.88]
	JHFT7	0.49	[0.075, 0.83]	0.23	[−0.15, 0.69]	0.39	[−0.02, 0.79]

**Figure A1:**
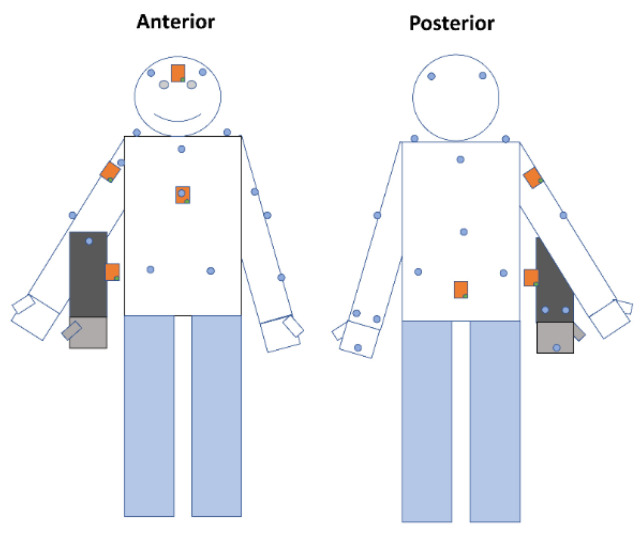
Illustration of sensor locations with the anterior view on the left and posterior view on the right. Orange boxes represent IMU sensors. Blue dots represent retroreflective markers.

## Data Availability

The code and data will be made publicly available at https://github.com/dbp-osel.

## Appendix A

The parent sensor is the sensor used to define a local coordinate system for a given body segment, which will be called the parent body segment. The child sensor is the sensor that measures the motion of a different body segment, which will be called the child segment. The method described below defines the motion of the child body segment relative to the local coordinate system defined by the parent sensor as measured through Euler decomposition.

**Table A1:** Definitions of Parent and Child Segments for each joint of Interest.

Joint	Parent	Child
Elbow	Upper Arm	Forearm
Shoulder	Torso	Upper Arm
Neck	Torso	Head
Torso	Torso	Pelvis

First, the quaternion outputs of the parent and child sensors are transformed into standardized local coordinate systems. The known locations of each sensor upon the body are used to define three element vectors which correspond to the Superior-Inferior (SI), Medial-Lateral (ML), and Anterior-Posterior (AP) axes of the sensor that align with the body. These vectors can be called SICalVec, MLCalVec, APCalVec, respectively. These three element vectors are used to transform the coordinate systems of the sensors from sensor-based local coordinates to body-based local coordinates through quaternion conjugations.

The first step to determining the Euler angles is taking the standardized SI, ML, and AP axes of the child sensor. These axes are defined relative to the body of the participant, so the SI axis of the sensor corresponds to the SI axis of the participant’s body. The ML axis of the sensor corresponds to the ML axis of the body, and the AP axis corresponds to the AP axis of the body. These axes are used to produce the three vectors ProjectedVector_SI_, ProjectedVector_ML_, ProjectedVector_AP_, which are defined by projecting the SI, ML, and AP axes of the child sensor onto the standardized local coordinate system defined by the parent sensor, X-Y-Z.

The first rotation of the Euler decomposition, labeled Rotation_Y_, as it rotates the child sensor’s projected SI axis in the local coordinate system’s original Z-X plane, which is described as follows in Equation (A1). This rotation also results in a new interim coordinate system with axes labeled X′, Y′, Z′.

(A1) $${\mathrm{Rotation}}_{Y}=\tan^{-1}\left(\frac{{\mathrm{ProjectedVector}}_{\mathrm{SIComponent}3\;}}{{\mathrm{ProjectedVector}}_{\mathrm{SIComponent}1\;}}\right)$$

The second rotation of the Euler decomposition is performed about the new X’ axis that results from Equation (A1). This can be found in the Z′-Y plane, which is identical to the Z′-Y′ plane as Equation (A1) did not involve the local coordinate system’s *y*-axis. The ProjectedVector_SI_ component along the z’ axis can be found from the components of the original z-x plane using the Pythagorean theorem. This is used to define ProjectedVectors_SIComponentZ’_ in Equation (A2). ProjectedVectors_SIComponentZ’_ is then used to find the resultant second Euler rotation about the new X′ axis with Equation (A3). This also results in a new coordinate system with axes labeled X″, Y″, Z″.

(A2) $${\mathrm{ProjectedVector}}_{\mathrm{SIComponent}Z^{\prime }}=\surd \left({\mathrm{ProjectedVector}}_{\mathrm{SIComponent}1}^{2}+{\mathrm{ProjectedVector}}_{\mathrm{SIComponent}3\;}^{2}\right)$$

(A3) $${\mathrm{Rotation}}_{X^{\prime }}=\tan^{-1}\left(\frac{{\mathrm{ProjectedVector}}_{\mathrm{SI}\;\mathrm{Component}2}}{{\mathrm{ProjectedVector}}_{\mathrm{SIComponent}Z^{\prime }}}\right),$$

To determine the third Euler Angle about Z″, first a quaternion is defined (A4) to describe rotation about axis Y from the X, Y, Z coordinate system, also known as the ML axis. MLCalVec is a three-element vector describing the ML axis of the parent sensor, which was pre-defined by the known orientations of the individual IMU sensors on the body. This vector is the same vector previously used to transform the individual sensor coordinate systems into uniform body-based coordinate systems.

(A4) $$\begin{array}{l} {\mathrm{Quaternion}}_{\mathrm{rotationY}}=\\ {}[\cos\left(\frac{{\mathrm{Rotation}}_{y}}{2}\;\right);\;\sin\left(\frac{{\mathrm{Rotation}}_{y}}{2}\right)\times {\mathrm{MLCalVec}}_{\mathrm{component}1};\sin\left(\frac{{\mathrm{Rotation}}_{y}}{2}\right)\\ \;\times {\mathrm{MLCalVec}}_{\mathrm{component}2};\;\sin\left(\frac{{\mathrm{Rotation}}_{y}}{2}\right)\times {\mathrm{MLCalVec}}_{\mathrm{component}3}] \end{array}$$

Quaternion_rotationY_ is then used to rotate the X axis from the X, Y, Z coordinate system to find the X’ axis through quaternion conjugation as described in (A5). The X’ Axis vector is then projected onto the coordinate system defined by ProjectedVector_SI_, ProjectedVector_ML_, and ProjectedVector_AP_ to produce ProjectedX’Axis. The third Euler Angle about Z’’ is found with the components of ProjectedX’Axis as described in (A6).

(A5) $$X^{\prime }\mathrm{Axis}=\left({\mathrm{Quaternion}}_{\mathrm{rotationY}}\times {\mathrm{Quaternion}}_{\mathrm{XAxis}}\right)\times {\mathrm{Quaternion}}_{\mathrm{rotationY}}^{-1}$$

(A6) $${\mathrm{Rotation}}_{Z^{\prime \prime }}=\tan^{-1}({\mathrm{ProjectedX}}^{\prime }{\mathrm{Axis}}_{\mathrm{Component}2}/{{\mathrm{ProjectedX}}^{\prime }\mathrm{Axis}}_{\mathrm{component}3}\;$$

## Appendix B

The sensors of the three systems were not expected to interfere due to the independent locations of the sensor placements and the differences in the recording mechanisms. Excepting the sternum location alone, all other IMUs and retroreflective markets were mounted directly on the body (Figure A1). The sternum location required the sternum reflective marker to be mounted on the sternum IMU sensor for the tracking accuracy of the Vicon software.

During the data cleaning and processing phase, review of the exported data showed that the markerless system’s software and background subtraction mechanism detected neither the retroreflective markers used by the optical system-based system nor the IMU sensors. The IMU sensors were not retroreflective and were not detected by the optical marker-based system.
